# Identification of a metastatic lung adenocarcinoma of the palate mucosa through genetic and histopathological analysis: a rare case report and literature review

**DOI:** 10.1186/s12885-019-5277-1

**Published:** 2019-01-11

**Authors:** Masanobu Abe, Kousuke Watanabe, Aya Shinozaki-Ushiku, Tetsuo Ushiku, Takahiro Abe, Yuko Fujihara, Yosuke Amano, Liang Zong, Cheng-Ping Wang, Emi Kubo, Ryoko Inaki, Naoya Kinoshita, Satoshi Yamashita, Daiya Takai, Toshikazu Ushijima, Takahide Nagase, Kazuto Hoshi

**Affiliations:** 1 Department of Oral & Maxillofacial Surgery, University of Tokyo Hospital, 7-3-1 Hongo, Bunkyo-ku, Tokyo, 113-8655 Japan; 2 Division for Health Service Promotion, University of Tokyo, Tokyo, Japan; 3 Department of Respiratory Medicine, University of Tokyo Hospital, 7-3-1 Hongo, Bunkyo-ku, Tokyo, 113-8655 Japan; 4 Department of Pathology, Graduate School of Medicine, the University of Tokyo, Tokyo, Japan; 5 Graduate School of Medicine, the University of Tokyo, Tokyo, Japan; 6 Department of Gastrointestinal Surgery, Peking University Cancer Hospital & Institute, Beijing, China; 7 Department of Otolaryngology, National Taiwan University Hospital, Taipei, Taiwan; 8 Division of Epigenomics, National Cancer Center Research Institute, Tokyo, Japan; 9 Department of Clinical Laboratory, University of Tokyo Hospital, Tokyo, Japan

**Keywords:** Cancer of unknown primary origin (CUP), Occult primary tumor, Lung adenocarcinoma, Minor salivary gland tumor (MSGT), Metastatic cancer

## Abstract

**Background:**

Cancers of unknown primary origin (CUPs) are reported to be the 3-4th most common causes of cancer death. Recent years have seen advances in mutational analysis and genomics profiling. These advances could improve accuracy of diagnosis of CUPs and might improve the prognosis of patients with CUPs.

**Case presentation:**

A 76-year old male with an adenocarcinoma of unknown primary origin in the lung presented with another tumor of the palate mucosa. The tumor cells in the pleural effusion were all negative for immunohistochemical markers (TTF-1 and Napsin A) and lung-specific oncogenic driver alterations (*EGFR* mutation and *ALK* translocation). The tumor of the palate mucosa was likewise identified as an adenocarcinoma, and the cells showed cytological similarities with the tumor cells in the pleural effusion; TTF-1, Napsin A, *EGFR* mutation and *ALK* translocation were all negative. This result suggested that origins of the tumors of the palate mucosa and in the lung were the same, even though the origin had not yet been determined. Next, we addressed whether the tumor of the palate mucosa was a primary tumor or not. Secretory carcinoma (SC), which is a common type of minor salivary gland tumor (MSGT), was suspected; however, mammaglobin was negative and *ETV6-NTRK3 (EN)* fusion was not observed. Other MSGTs were excluded based on histological and immunohistochemical findings. Furthermore, an additional examination demonstrated an oncogenic *KRAS* mutation at codon 12 (p.G12D) in both palate tumor and in pleural effusion. *KRAS* mutation is known to exist in one-third of lung adenocarcinomas (LUADs), but quite rare in MSGTs. The possibility of metastasis from other organs was considered unlikely from the results of endoscopic and imaging studies. This result indicated that the primary site of the CUP was indeed the lung, and that the tumor of the palate mucosa was a metastasis of the LUAD.

**Conclusions:**

A tumor of the palate mucosa that showed diagnostic difficulties was determined to be a metastatic LUAD by genomic alterations and histopathological findings.

**Electronic supplementary material:**

The online version of this article (10.1186/s12885-019-5277-1) contains supplementary material, which is available to authorized users.

## Background

Carcinomas of unknown primary origin (CUPs) comprise a heterogeneous group of cancers for which the site of origin remains occult after detailed investigations [1]. CUPs are the 3-4th most common causes of cancer death [2]. Accurate diagnosis and effective therapy is important to improve the poor prognosis. Recent progress in analytical technologies is allowing CUPs to be characterized by genetic information [3, 4].

The most common malignant neoplasms of the palate mucosa are known to be minor salivary gland tumors (MSGTs) such as adenoid cystic carcinoma (AdCC), mucoepidermoid carcinoma (MEC), and secretory carcinoma (SC), followed by squamous cell carcinoma (SCC) and malignant melanomas (MM) [5–9]. On the other hands, metastatic tumors to the oral cavity from a distant organ is uncommon. It represents approximately 1–3% of all oral malignancies. Such metastases can occur to the bone or to the oral soft tissues [10]. Almost any malignancy from any site is capable of metastasis to the oral cavity even though the rate is quite low. The most common primary malignancies presenting oral metastases are the lung, kidney, liver, and prostate for men, and breast, genital organs, kidney, and colorectum for women [11].

In this case study, we addressed an adenocarcinoma of unknown primary origin of the palate mucosa and identified it as a metastatic lung adenocarcinoma (LUAD) by both genetic and histopathological analytic approaches [12, 13].

## Case presentation

**Fig. 1:**
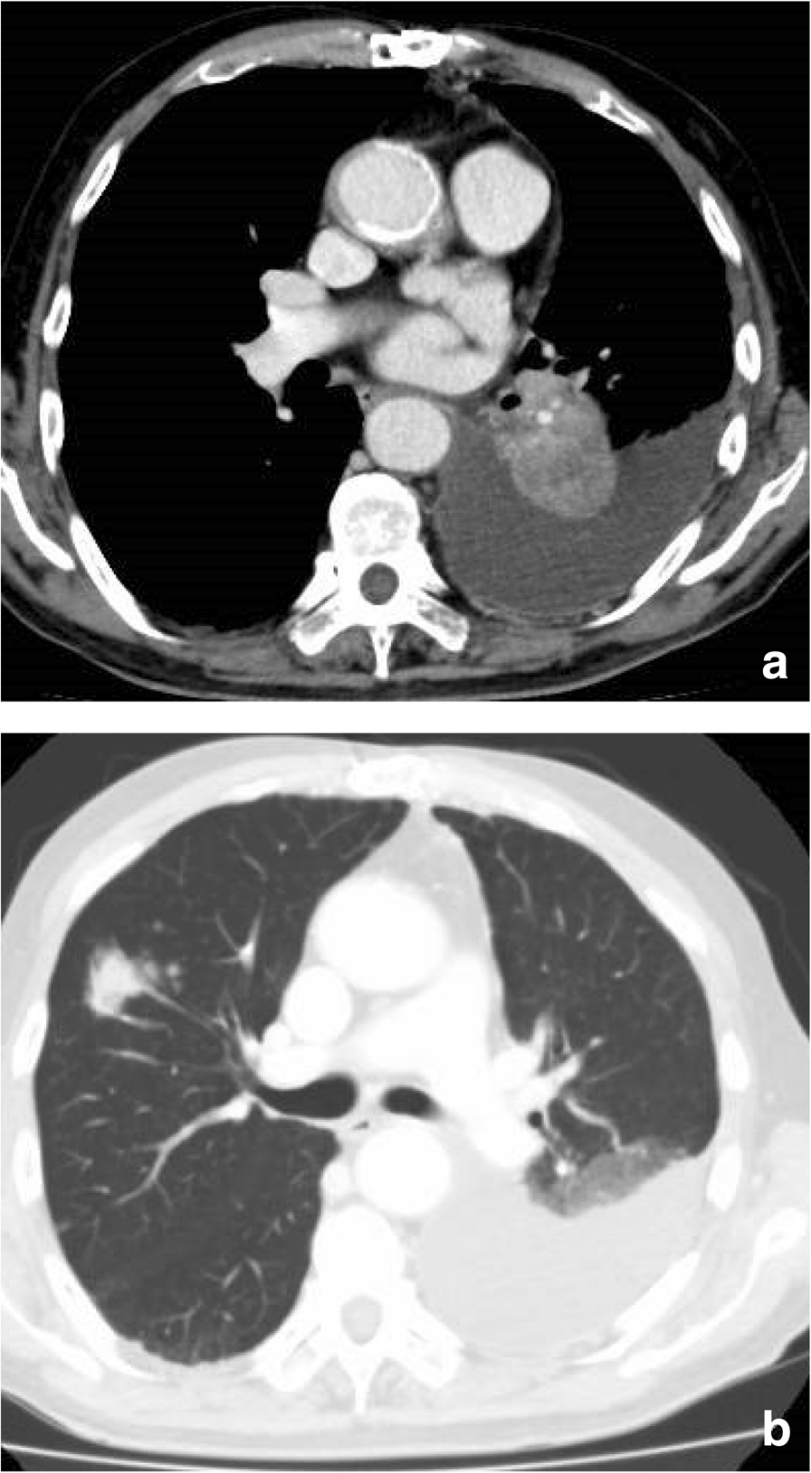
CT scan of the chest. CT scan of the chest, showing left hilar lung mass and left pleural effusion (**a**) and multiple lung nodules in the right lung (**b**)

**Fig. 2:**
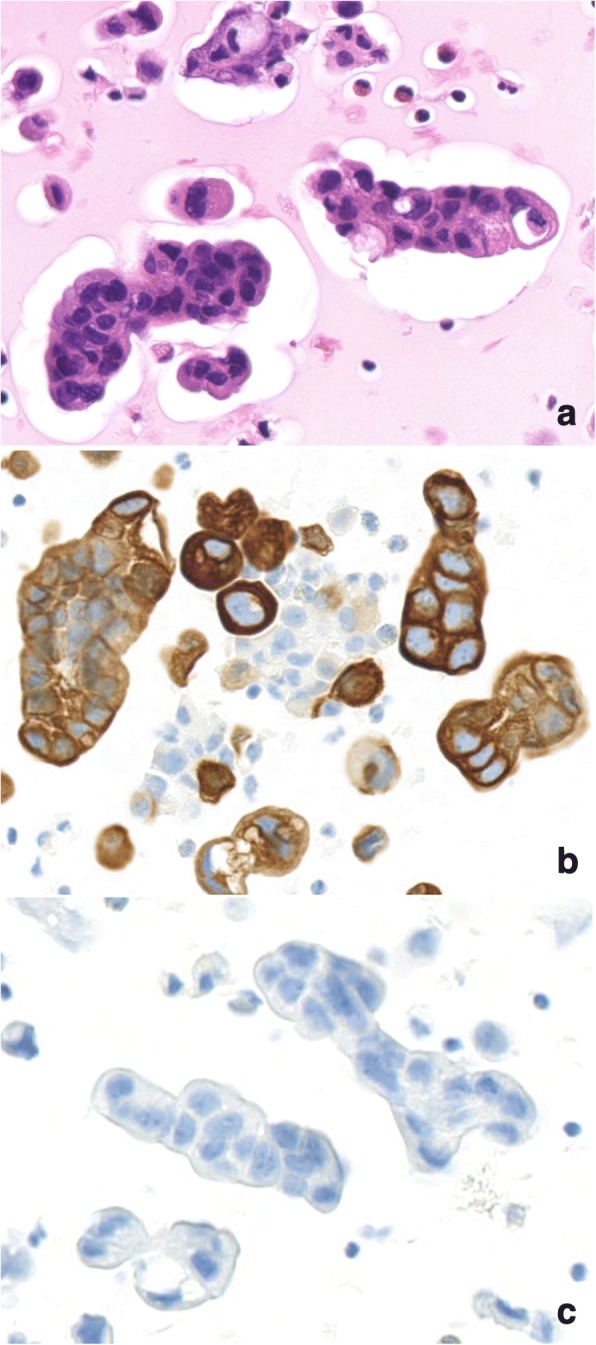
Cytological diagnosis using cell block samples from pleural effusion. **a** Hematoxylin and eosin (HE) staining at a 400× magnification. **b** Positive staining of cytokeratin 7 (CK7). **c** Negative staining for thyroid transcription factor 1 (TTF-1)

A 76-year old male presenting as a one-month history of dry cough and left chest pain was admitted to our hospital. The patient had a past history of aortic stenosis, abdominal aortic aneurysm, and chronic atrial fibrillation, and he had smoked one and a half pack of cigarettes per day for 27 years from the age of 20 to 47. CT scan of the chest showed left hilar lung mass, left pleural effusion, atelectasis of the left lower lobe and multiple lung nodules predominantly in the right lung (Fig. 1). Cytological examination of the pleural effusion revealed adenocarcinoma cells and immunohistochemistry (IHC) analysis of pleural effusion cell block was performed to determine the primary organ from which the cancer developed. Malignant cells in the pleural effusion were positive for Cytokeratin 7 (CK7) and negative for cytokeratin 20 (CK20) (Fig. 2). These cells were negative for two lung adenocarcinoma (LUAD) markers; TTF1 and Napsin A, and IHC analysis could not determine the primary organ of the tumor. Adenocarcinoma cells in the pleural effusion were also negative for LUAD specific oncogenic driver mutations: *EGFR* mutation and *ALK* translocation determined using the PCR-invader method [11] and the intercalated antibody-enhanced polymer (iAEP) method (HISTOFINE *ALK* iAEP® kit, Nichirei Biosciences, Inc., Tokyo, Japan) [12], respectively. The values of serum tumor markers were as follows: CEA 2.9 ng/ml (normal range, 0 to 5); CA19–92326 U/ml (normal range, 0 to 37); CYFRA 57.7 ng/ml (normal range, 0 to 3.5); pro-GRP 34.5 pg/ml (normal range, 0 to 80.9); PSA 0.96 ng/ml (normal range, 0 to 4). Although the primary organ was not clear, the patient was treated by the combination of carboplatin (AUC 5) and paclitaxel (200 mg/m2), which is one of the standard chemotherapy for both LUAD and CUP.

**Fig. 3:**
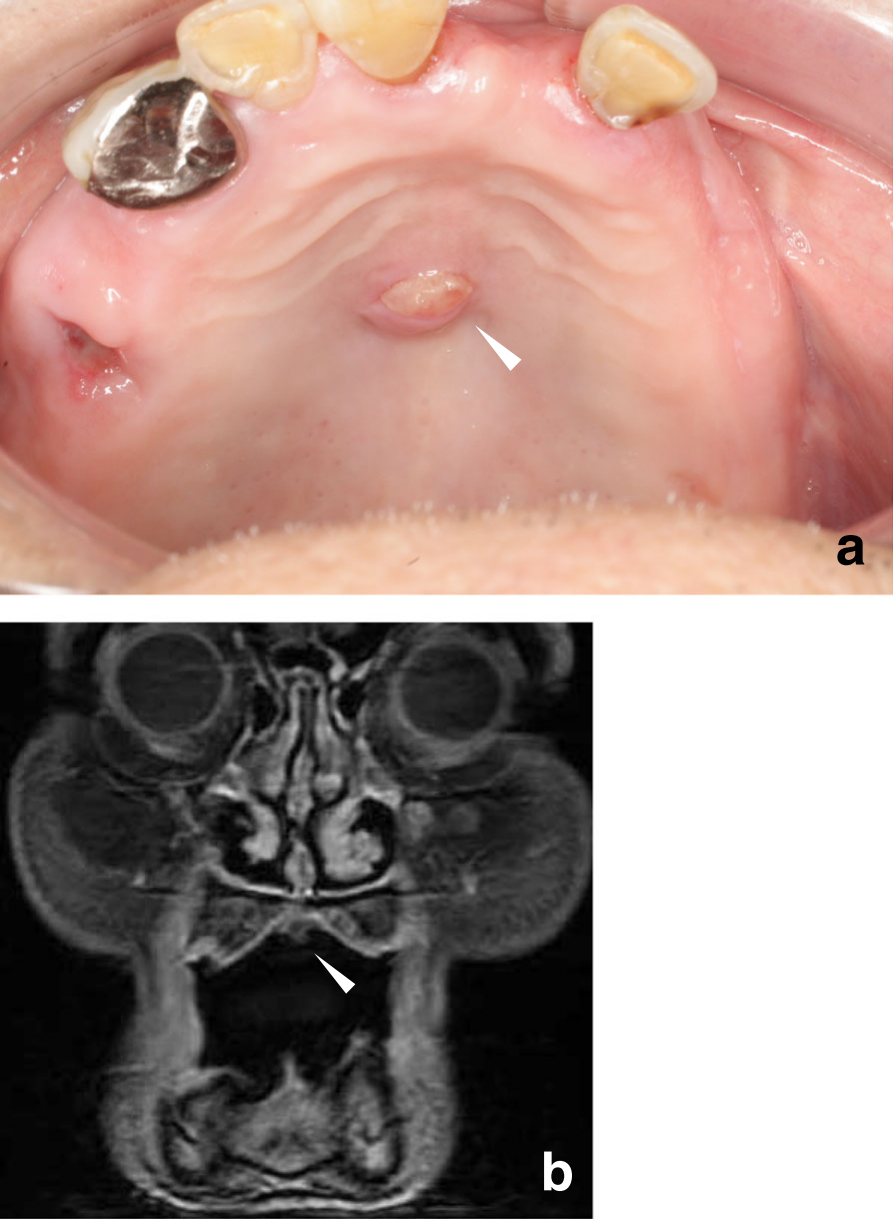
Clinical findings of the mass of the palate mucosa. **a** The arrowhead showed a mass of the palate mucosa. It was a small (major axis; 7 mm) and round mass with smooth surface (arrowhead). **b** Magnetic Resonance Imaging (MRI) showed a small mass localized in the palate mucosa (arrowhead)

A tumor of the palate mucosa was noticed on physical examination of the oral cavity. The tumor of the palate mucosa was a small (major axis; 7 mm) and round mass with smooth surface. It located in the middle of his palate. Magnetic Resonance Imaging (MRI) showed this tumor in the palate; however, deep invasion was not observed (Fig. 3). ^18^F-Fluorodeoxyglucose-positron emission tomography/computed tomography (FDG-PET/CT) indicated abnormal intake of FDG of the palate mucosa and both lungs, which were considered malignant lesions (Additional file 1: Figure S1). Multiple lymph node metastases, multiple bone metastasis, and pleural dissemination were also suspected.

**Fig. 4:**
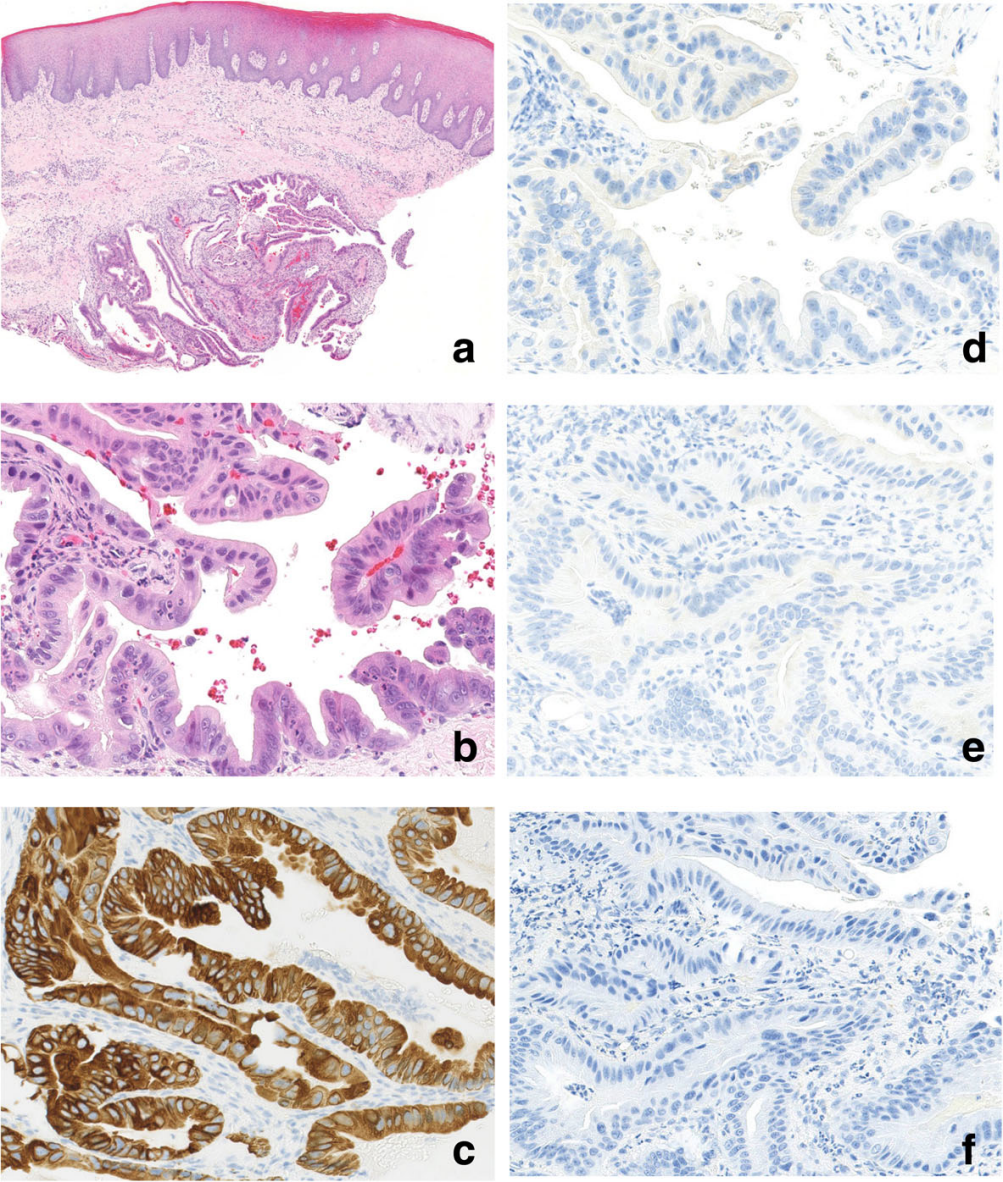
Histopathological findings for the mass of the palate mucosa. HE staining image with a loupe (**a**) and at a 200× magnification (**b**). An adenocarcinoma consisting of tubular or papillary proliferation of columnar-shaped tumor cells invading the subepithelial tissue. The tumor cells were positive for CK7 (**c**) and negative for TTF-1 (**d**), mammaglobin (**e**) and S-100 (**f**)

A biopsy was performed for the tumor of the palate mucosa under the local anesthesia. The histology revealed an adenocarcinoma consisting of tubular or papillary proliferation of columnar-shaped tumor cells invading the subepithelial tissue (Fig. 4). The tumor cells were positive for CK7 and negative for CK20, TTF-1 and Napsin A, which was consistent with the result of the pleural effusion. Whether the tumor of the palate mucosa was a metastatic or primary tumor remained inconclusive at this time.

Then, we evaluated the possibility of this tumor in the palate mucosa as a primary tumor. Most common malignant neoplasms of the palate mucosa are known to be MSGTs. Especially, SC is one of the common MSGT [6], however mammaglobin and S-100 protein was immunohistochemically negative and *ETV6-NTRK3 (EN)* fusion was not observed in fluorescence in situ hybridization (FISH) analysis by using Vysis®LSI® ETV6 Break Apart Rearrangement Probe (Abbott Molecular/Vysis) (Additional file 2: Figure S2). Other MSGT such as AcCC, AdCC, PLGA, and MEC were also excluded as a diagnosis based on histological and immunohistochemical findings.

**Fig. 5:**
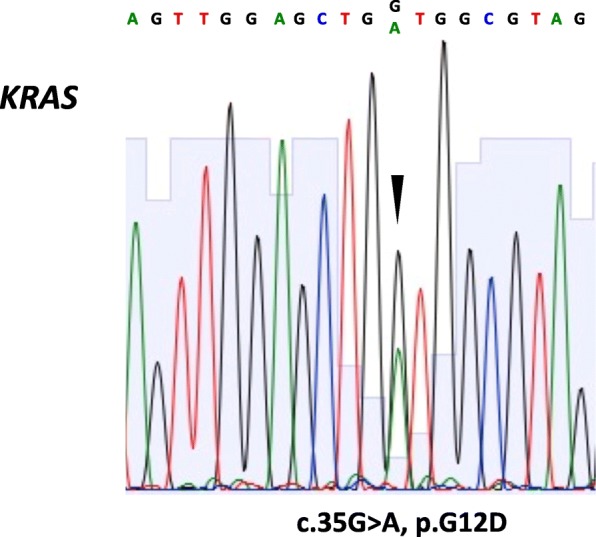
*KRAS* mutation in pleural effusion. Oncogenic *KRAS* mutation at codon 12 (p.G12D) was identified in pleural effusion. The missense mutation (c.35G > A) is indicated by arrowhead

Because of the absence of *EGFR* mutation and *ALK* translocation, this case was registered to Lung Cancer Genomic Screening Project for Indivisualized Medicine in Japan (LC-SCRUM-Japan). The cancer genome screening of the fresh frozen tumor of the palate mucosa was performed using Oncomine® Cancer Research Panel (OCP, Thermo Fisher Scientific, MA, USA), which successfully identified an oncogenic *KRAS* mutation at codon 12 (p.G12D). Furthermore, presence or abscense of *KRAS* mutation in pleural effusion was examined. Genomic DNA was purified from formalin-fixed paraffin-embedded (FFPE) cells of pleural effusion using Deparaffinization Solution (QIAGEN) and QIAamp DNA FFPE Tissue Kit (QIAGEN). PCR was performed using 40 ng genomic DNA and the following primers; forward primer, 5′- AGGCCTGCTGAAAATGACTG -3′, and reverse primer, 5′- GGTCCTGCACCAGTAATATGCA -3′ (annealing temperature: 55 °C) [14]. As a result, *KRAS* mutation at codon 12 (p.G12D) was also identified in pleural effusion by direct sequencing (Fig. 5). The *KRAS* mutation is known to exist in one third of the LUAD, but it is quite rare in MSGT [15, 16]. Although the *KRAS* mutation is also known to be one of the common abnormalities in pancreatic and colorectal cancers [17], the possibility of metastasis from colorectal cancer is quite unlikely because gastrointestinal endoscopy did not show the presence of malignant lesions. The metastasis of pancreatic cancer is also unlikely from the results of CT scan and FDG-PET/CT. Together with the cytological similarities between tumor cells in the pleural effusion and those of the palate mucosa, we concluded that the tumor of the palate is a metastatic stage IV LUAD (cT3N3M1c according to the 8th edition of TNM staging of lung cancer). Adenocarcinoma, not otherwise specified (NOS), that shows glandular or ductal differentiation but lacks the prominent histomorphologic features was excluded as a diagnosis because the carcinoma in this study was characterized other, more specific types of carcinoma.

The disease progressed after two cycles of chemotherapy with carboplatin and paclitaxel. The patient received two cycles of immunotherapy with nivolumab as a second line therapy, but died due to disease progression four months after the first admission.

## Discussion and conclusions

CUP is a heterogeneous group of cancers for which the anatomical site of origin remains obscure despite detailed evaluation [18, 19]. CUPs account for 3–5% of all malignant epithelial tumors and, importantly, are the 3-4th most common causes of cancer death [2, 19]. Management of CUPs requires a thorough physical examination, imaging test and pathologic review [20]. Site-specific therapy can be selected when a putative primary site is identified. Otherwise, empiric chemotherapy is adopted [18]. However, survival outcomes in CUP patients remain poor [21]. To ensure that patients with CUP can receive optimal care, identification of genetic abnormalities in addition to existing surveillance is urgently needed [2, 20, 22].

In the head and neck (HN) region, it was reported that 1% of malignant solid tumors were metastatic cancers from distant primary sites. Sagheb et al. reported that CUPs accounted for more than 20% of metastatic cancers in the HN region (HNCUPs) [23]. Overgaard et al. and Lanzer et al. found that 1.5 and 8.9% of CUPs were located in HN regions, respectively [24, 25]. Balaker et al. reported that survival outcomes of patients with HNCUPs were most significantly influenced by clinical stage at the time of diagnosis and that treatment modalities did not affect the survival outcomes [26]. For SCCs of HNCUPs, the role of human papillomavirus (HPV) infection is a current topic. Sivars et al. indicated that HPV was a diagnostic and prognostic factor in HNCUPs [27–29]. *p16,* an important tumor suppressor gene in cancers [30], is also known as a surrogate marker of HPV infection. Dixon et al. reported that *p16*-positive status was an independent predictor of disease-free survival (DFS) for patients with HNCUPs histologically diagnosed to be SCCs [31]. Schroeder et al. emphasized that HPV status should be included in HNCUP diagnosis and in therapeutic decision-making [32]. By contrast, the number of reports about HNCUPs histologically diagnosed as adenocarcinomas is quite limited.

In this study, a CUP of the palate mucosa was clarified to be metastatic lung cancer through genetic and histopathological approaches. Lung cancer is known to be the leading cause of cancer deaths worldwide. NSCLC, constituting more than 80% of all lung cancers, is a heterogeneous disease with multiple different oncogenic driver mutations [15, 33–35]. In adenocarcinomas with defined alterations such as *EGFR* mutations and *ALK* translocations, targeted therapies are now the first-line standard of care [36]. *KRAS* represents one of the most common oncogenic driver mutations in human cancers; however, targeted therapies have not been available yet [33, 37]. In contrast to *EGFR* mutation and *ALK* translocations that are frequently observed in non-smokers, *KRAS* mutation in lung cancer is prevalent in male smokers [38], which is consistent with the present case.

As 10 to 20% cases of LUAD are negative for TTF-1 and Napsin A [38], *KRAS* mutation testing is sometimes useful to determine the primary organ of the tumor as shown in the current study. *KRAS* mutation is frequently observed in lung, pancreatic and colorectal cancers [17]. In salivary grand cancer, two sarcomatoid salivary duct carcinomas were reported to show *KRAS* mutations (A146T and Q61H) [39]. However, *KRAS* mutation is quite rare in MSGTs. Only one case of AdCC with a GGT-GAT transition at codon 12 (Gly12Asp) has been reported [16]. In MSGTs, driver fusion genes have already been elucidated; *ETV6-NTRK3* in SC, *MYB-NFIB* in AdCC, *CTRC1-MAML2, CTRC3-MAML2, EWSR1-POU5F1 i*n MEC.

Although the target therapy for *KRAS* has not been established, the *KRAS* mutation testing is important not only for diagnosis but also for determination of therapeutic strategy. In colorectal cancer, *KRAS* mutation testing is widely used in clinical practice to predict the response to anti-*EGFR* monoclonal antibody therapy [40]. *KRAS* mutation testing in lung cancer has not yet been established in clinical routines, but recent studies suggest its value as predictive biomarker [41]. A meta-analysis showed that *KRAS* mutation may be a marker for survival benefits to immune checkpoint inhibitors [42]. In this case, however, the immunotherapy with nivolumab was not effective. Further evidence is required to use *KRAS* testing routinely as a predictive biomarker for lung cancer.

In conclusion, an adenocarcinoma of unknown primary origin in the palate mucosa was determined to be a rare case of metastatic LUAD by genomic alterations and histopathological findings.

## Additional files

Additional file 1: **Figure S1.** Detection of malignant lesions using ^18^F-Fluorodeoxyglucose-positron emission tomography/computed tomography (FDG-PET/CT). Abnormal intake of FDG was indicated in the middle of the palate (a) and both lungs (b). (PPTX 147 kb)

Additional file 2: **Figure S2.** Fluorescence in situ hybridization (FISH) analysis of *ETV6* gene rearrangement. *ETV6-NTRK3 (EN)* fusion was not observed. The arrowheads show representative cells without *EN* fusion. (PPTX 304 kb)

## Abbreviations

AdCC: Adenoid cystic carcinoma
CK20: Cytokeratin 20
CK7: Cytokeratin 7
CUP: Cancers of unknown primary origin
DFS: Disease-free survival
*EN*: *ETV6-NTRK3*
FDG-PET/CT: ^18^F-Fluorodeoxyglucose-positron emission tomography/computed tomography
FISH: Fluorescence in situ hybridization
HN: Head and neck
HPV: Human papillomavirus
iAEP: Intercalated antibody-enhanced polymer
IHC: Immunohistochemistry
LUADs: Lung adenocarcinomas
MEC: Mucoepidermoid carcinoma
MM: Malignant melanomas
MSGT: Minor salivary gland tumor
SC: Secretory carcinoma
SCC: Squamous cell carcinoma
