# Supplementary figures and images for: Identification of a metastatic lung adenocarcinoma of the palate mucosa through genetic and histopathological analysis: a rare case report and literature review
Source: BMC Cancer. 2019 Jan 11;19:52. doi: 10.1186/s12885-019-5277-1 (PMC6329170; doi:10.1186/s12885-019-5277-1)

## Slide 1
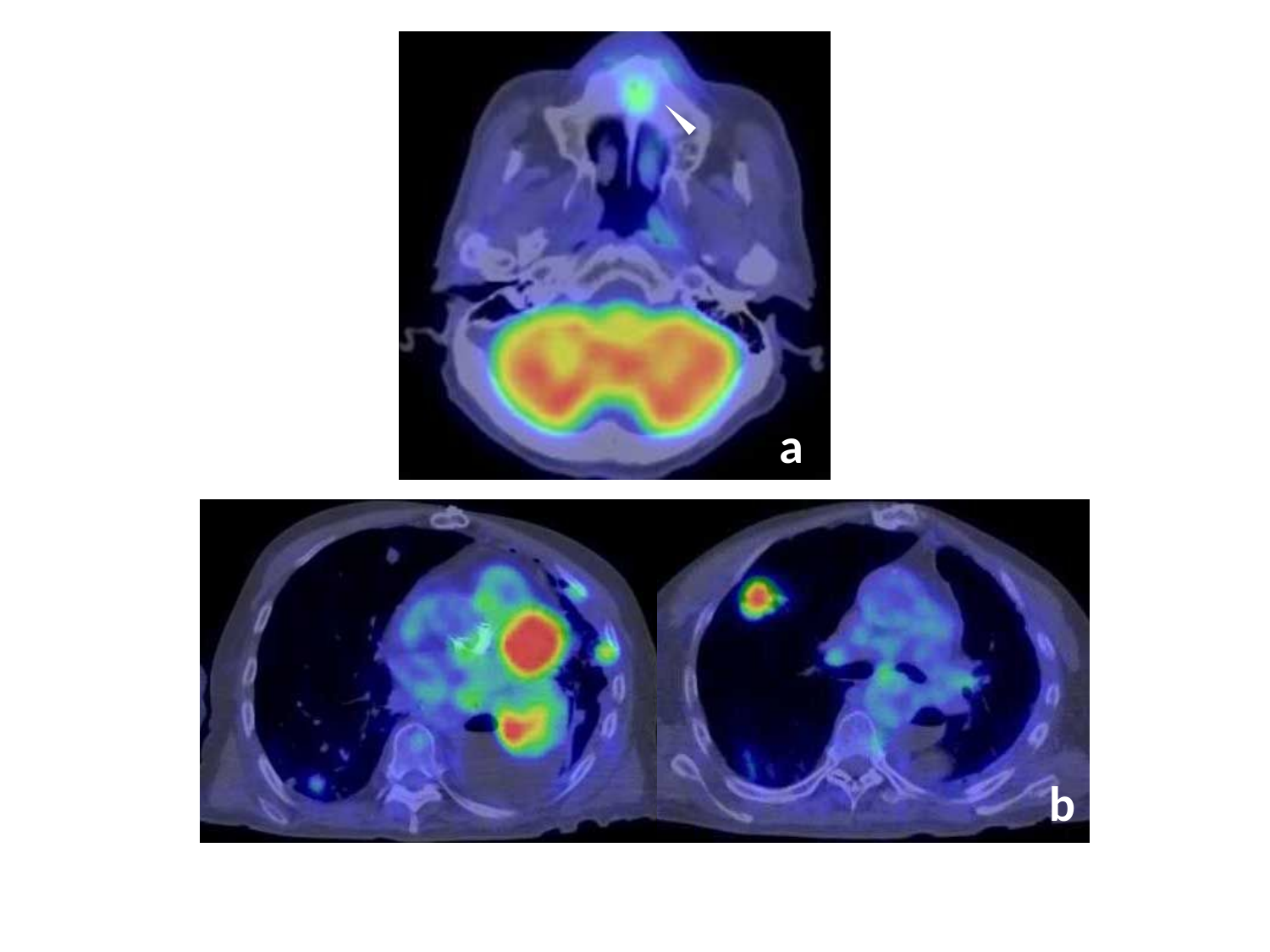

a
b
# Figure S1

Supplement: Supplementary file 1 — Figure S1. Detection of malignant lesions using 18F-Fluorodeoxyglucose-positron emission tomography/computed tomography (FDG-PET/CT). Abnormal intake of FDG was indicated in the middle of the palate (a) and both lungs (b). (PPTX 147 kb) [file 12885_2019_5277_MOESM1_ESM.pptx]

## Slide 1
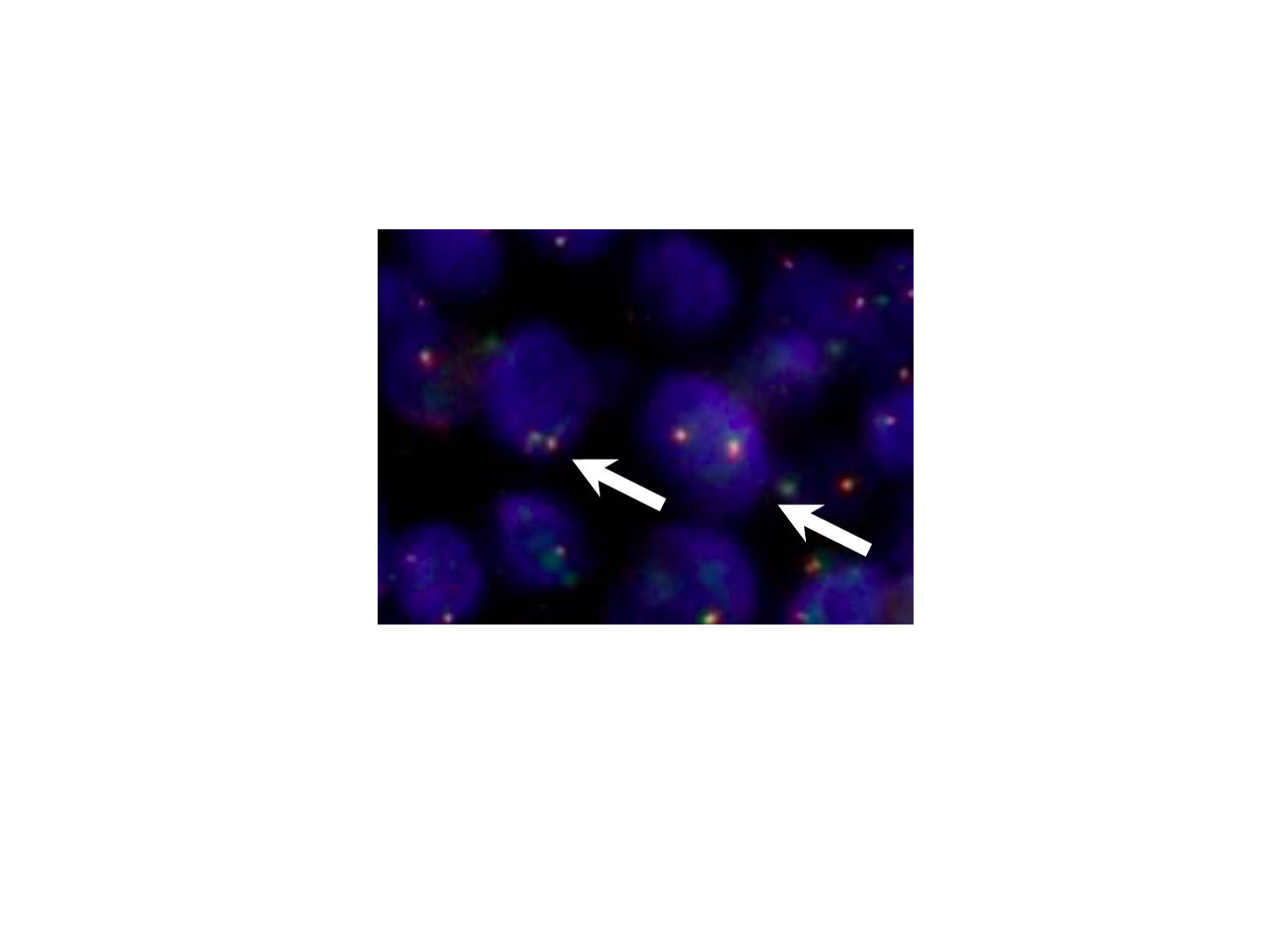

# Figure S2

Supplement: Supplementary file 2 — Figure S2. Fluorescence in situ hybridization (FISH) analysis of ETV6 gene rearrangement. ETV6-NTRK3 (EN) fusion was not observed. The arrowheads show representative cells without EN fusion. (PPTX 304 kb) [file 12885_2019_5277_MOESM2_ESM.pptx]
